# Korean species of *Aleochara* Gravenhorst subgenus *Xenochara* Mulsant & Rey (Coleoptera, Staphylinidae, Aleocharinae)

**DOI:** 10.3897/zookeys.60.404

**Published:** 2010-10-07

**Authors:** Jong-Seok Park, Kee-Jeong Ahn

**Affiliations:** 1 Department of Entomology, Louisiana State University Agricultural Center, Baton Rouge, LA, 70803, USA; 2 Department of Biology, Chungnam National University, Daejeon 305-764, Republic of Korea

**Keywords:** Aleocharini, Aleochara, Xenochara, redescription, Korea

## Abstract

A taxonomic review of Aleochara Gravenhorst subgenus Xenochara Mulsant & Rey in Korea is presented. Five species are recognized, with one species, Aleochara (Baryodma) intricata Mannerheim, newly transferred to the subgenus Xenochara. Aleochara (Xenochara) asiatica Kraatz and Aleochara (Xenochara) peninsulae Bernhauer are reported for the first time in the Korean peninsula. A key, line drawings of diagnostic characters, and redescriptions of Korean Xenochara species are provided.

The staphylinid genus Aleochara Gravenhorst includes over 400 species in 19 subgenera worldwide. Fourteen species in six subgenera are recorded in the Korean peninsula (Smetana 2004; Park and Ahn 2009). The subgenera of Aleochara have been previously diagnosed using a few characters of the antenna, mesosternal carina, pronotal pubescence and microsculpture, elytral pubescence, maxillary palpi, and genitalia (Klimaszewski 1984). However, the morphologically diverse subgenera such as Xenochara are poorly distinguished from other Aleochara subgenera by these characters (Klimaszewski 1984).

Therefore, we used characters that are more informative at the subgeneric and species levels. Our character analysis follows the methods of Sawada (1972) and Ashe (1984). The terminology for abdominal segments follows Thayer (2005).

We redescribe five Korean Xenochara species herein, and a key and line drawings of diagnostic characters of these species are also provided. The Korean specimens studied are deposited in the Chungnam National University Insect Collection (CNUIC), Daejeon, Korea.

## Taxonomy

### Subgenus Xenochara

Mulsant & Rey

Xenochara Mulsant and Rey 1874: 60; Ganglbauer 1895: 32; Fenyes 1920: 403; Bernhauer and Scheerpeltz 1926: 781; Palm 1972: 426; Seevers 1978: 137.
Polychara Mulsant and Rey 1874: 64; Ganglbauer 1895: 34; Fenyes 1920: 408; Bernhauer and Scheerpeltz 1926: 785; Portevin 1929: 237; Seevers 1978: 136.
Isochara Bernhauer 1901: 440, 461.
Xenochara  See Klimaszewski (1984) for complete synonymy and references.

#### Type species:

Aleochara decorata Aubé.

#### Diagnosis.

**Figure 1.**
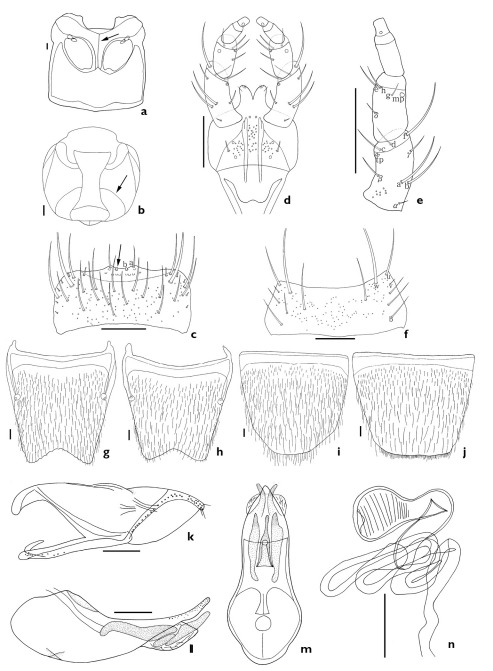
Aleochara (Xenochara) asiatica. **a** meso- and metaventrite, ventral aspect **b** head, ventral aspect **c** labrum, dorsal aspect **d** labium, ventral aspect **e** labial palpus, ventral aspect **f** mentum, ventral aspect **g** male tergite VIII, dorsal aspect **h** female tergite VIII, dorsal aspect **i** male sternite VIII, ventral aspect **j** female sternite VIII, ventral aspect **k** paramere, lateral aspect **l** median lobe, lateral aspect **m** median lobe, dorsal aspect **n** spermatheca. Scale bars = 0.1 mm.

**Figure 2.**
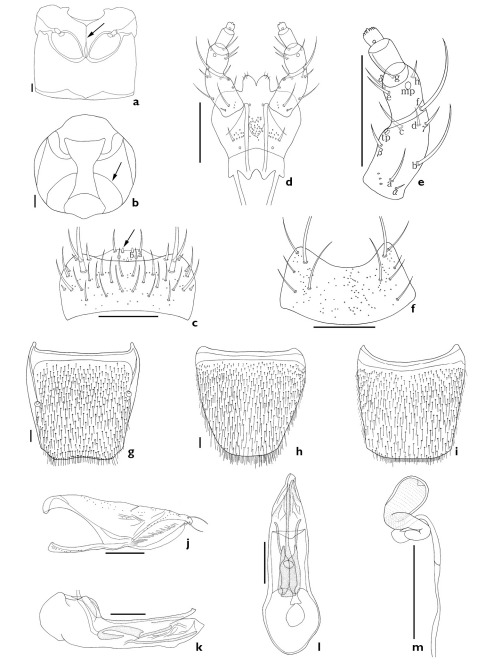
Aleochara (Xenochara) intricata. **a** meso- and metaventrite, ventral aspect **b** head, ventral aspect **c** labrum, dorsal aspect **d** labium, ventral aspect **e** labial palpus, ventral aspect **f** mentum, ventral aspect **g** tergite VIII, dorsal aspect **h** male sternite VIII, ventral aspect **i** female sternite VIII, ventral aspect **j** paramere, lateral aspect **k** median lobe, lateral aspect **l** median lobe, dorsal aspect **m** spermatheca. Scale bars = 0.1 mm.

**Figure 3.**
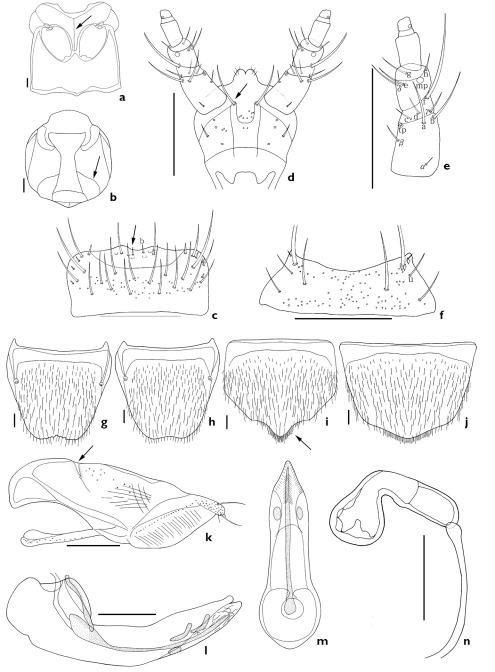
Aleochara (Xenochara) peninsulae. **a** meso- and metaventrite, ventral aspect **b** head, ventral aspect **c** labrum, dorsal aspect **d** labium, ventral aspect **e** labial palpus, ventral aspect **f** mentum, ventral aspect **g** male tergite VIII, dorsal aspect **h** female tergite VIII, dorsal aspect **i** male sternite VIII, ventral aspect **j** female sternite VIII, ventral aspect **k** paramere, lateral aspect **l** median lobe, lateral aspect **m** median lobe, dorsal aspect **n** spermatheca. Scale bars = 0.1 mm.

**Figure 4.**
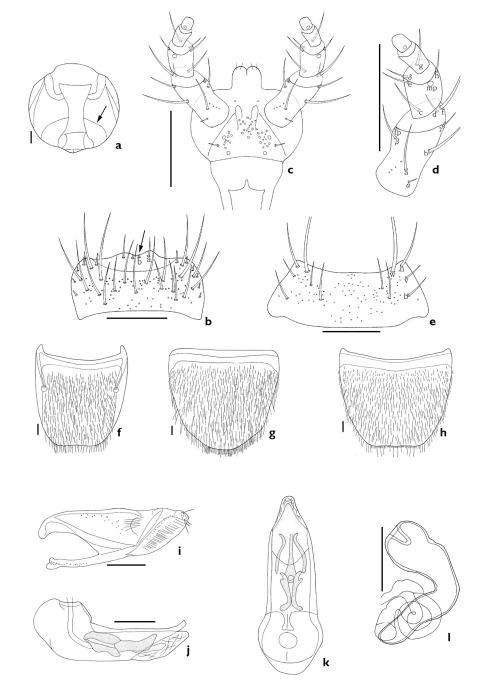
Aleochara (Xenochara) puberula. **a** head, ventral aspect **b** labrum, dorsal aspect **c** labium, ventral aspect **d** labial palpus, ventral aspect e mentum, ventral aspect **f** tergite VIII, dorsal aspect **g** male sternite VIII, ventral aspect **h** female sternite VIII, ventral aspect **i** paramere, lateral aspect **j** median lobe, lateral aspect **k** median lobe, dorsal aspect **l** spermatheca. Scale bars = 0.1 mm.

**Figure 5.**
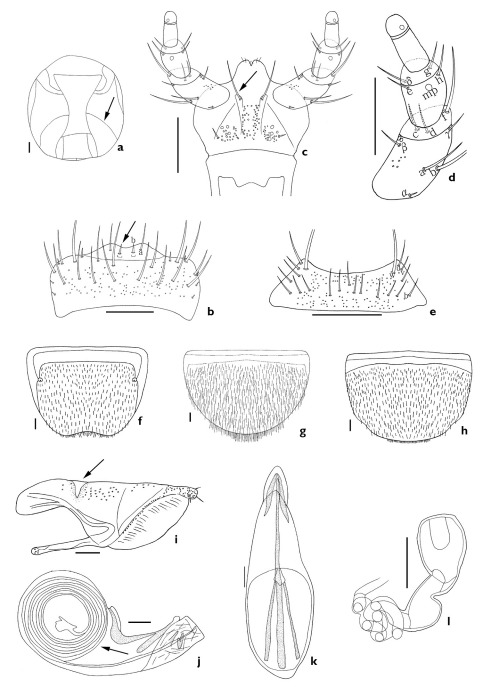
Aleochara (Xenochara) tristis. **a** head, ventral aspect **b** labrum, dorsal aspect **c** labium, ventral aspect **d** labial palpus, ventral aspect e mentum, ventral aspect **f** tergite VIII, dorsal aspect **g** male sternite VIII, ventral aspect **h** female sternite VIII, ventral aspect **i** paramere, lateral aspect **j** median lobe, lateral aspect **k** median lobe, dorsal aspect **l** spermatheca. Scale bars = 0.1 mm.

The subgenus Xenochara can be distinguished by a combination of the following characters: body compact, robust, pubescent; antennomere 4 usually longer than wide (except Aleochara tristis, transverse); carina on each side of midline of ventral surface of head present, attaining or almost attaining gular suture (arrows, Figs 1b, 2b, 3b, 4a, 5a; Klimaszewski 1984: Figs 313, 319, 321); maxillary palpomere 4 usually long (1/3 to 3/4 length of palpomere 3); labral b-seta sharpened or rounded apically (arrows, Figs 1c, 2c, 3c, 4b, 5b); mandibular internal tooth absent or weakly present; β-seta of labial palpi long (Figs 1e, 2e, 3e, 4d, 5d); mesoventrite completely or almost completely carinate (Figs 1a, 2a, 3a); pronotum evenly pubescent; spines of lateral margins of fore- and meso-tibia present but absent in meta-tibia.

#### Remarks.

This diagnosis is modified from Klimaszewski (1984: 35). New diagnostic characters based on mouthparts are added, and these are consistent at the subgenus level.

#### Key to the Aleochara (Xenochara) species from Korea

**Table:** 

1.	Elytra with emarginate latero-posterior margins	2
–	Elytra with rounded latero-posterior margins	3
2.	Labral b-seta acute (arrow, Fig. 3c), labium with a pair of distal setae (arrow, Fig. 3d), male abdominal tergite VIII with undulate posterior margin (secondary sexual dimorphism; Fig. 3g), male abdominal sternite VIII with produced posterior margin (secondary sexual dimorphism; arrow, Fig. 3i), paramere with fovea in hinge zone (arrow, Fig. 3k), spermathecal duct not coiled (Fig. 3n)	Aleochara peninsulae Bernhauer
–	Labral b-seta rounded at tip (arrow, Fig. 4b), labium without distal setae (Fig. 4c), male abdominal tergite VIII without undulate posterior margin (Fig. 4f), male abdominal sternite VIII with rounded posterior margin (Fig. 4g), paramere without fovea in hinge zone (Fig. 4j), spermathecal duct coiled (Fig. 4l)	Aleochara puberula Klug
3.	Antennomeres 5–6 longer than wide, abdominal tergite VIII with deeply emarginate posterior margin (Figs 1g, 1h), male tergite VIII with undulate posterior margin (secondary sexual dimorphism; Fig. 1g)	Aleochara asiatica Kraatz
–	Antennomeres 5–6 transverse, abdominal tergite VIII with weakly emarginate posterior margin (Figs 2g, 5f), male tergite VIII without undulate posterior margin (Figs 2g, 5f)	4
4.	Antennomere 4 longer than wide, labral b-seta rounded at tip (arrow, Fig. 2c), labium without distal setae (Fig. 2d), paramere without fovea in hinge zone (Fig. 2j), median lobe without coiled flagellum (Fig. 2k), spermathecal duct not coiled (Fig. 2m)	Aleochara intricata Mannerheim
–	Antennomere 4 transverse, labral b-seta acute (arrow, Fig. 5b), labium with a pair of distal setae (arrow, Fig. 5c), paramere with fovea in hinge zone (arrow, Fig. 5i), median lobe with coiled flagellum (arrow, Fig. 5j), spermathecal duct coiled (Fig. 5l)	Aleochara tristis Gravenhorst

#### Aleochara (Xenochara) asiatica

Kraatz

Fig. 1

Aleochara asiatica Kraatz 1859: 15; Bernhauer and Scheerpeltz 1926: 780; Pace 2001: 35 (mentioned as subgenus Xenochara); Smetana 2004: 356 (mentioned as subgenus Euryodma).
Aleochara japonica Sharp 1874: 8.
Aleochara (Isochara) asiatica Cameron 1939: 644.

##### Redescription.

Length 4.5–6.5 mm. Body large and robust; brownish black, antenna and legs black; elytra bicolored. Antennomeres 1–3 elongate, 4–6 longer than wide and 7–10 transverse. *Mouthparts*. Labrum transverse, bearing approximately 11 small, setae, and approximately 21 long setae, a-seta, b-seta, and pores present; b-seta rounded apically (arrow indicates b-seta, Fig. 1c). Labium with pseudopores in median area, approximately 2 real pores and pseudopores present in lateral area; a pair of basal pores present (Fig. 1d). Ligula with approximately 4 pairs of small setae apically (Fig. 1d). Labial palpi with large a-, b-, and f-seta of 12 setae present (a–h, α–δ); long β-seta present in the middle of palpomere 1; d-seta higher than c-seta (Fig. 1e). Mentum transverse, bearing 4 pairs of main setae (b, u, v, w), and 6 extra setae, and pores present (Fig. 1f). *Thorax*. Mesoventrite completely carinate (arrow, Fig. 1a). Elytra with round latero-posterior margin. *Abdomen*. Male and female abdominal tergite VIII with many short setae and pores; posterior margin deeply emarginate and undulate on male (Fig. 1g, h). Male and female abdominal sternite VIII with many short setae and pores, margin rounded and female with many small setae on posterior margin (Fig. 1i, j). *Genitalia*. Median lobe as in Figs 1l and m. Paramere without fovea in hinge zone (Fig. 1k). Spermatheca with duct coiled (Fig. 1n).

##### Material examined.

1♂, Daeheungsa-temple, Samsan-myeon, Haenam-gun, Jeonnam Prov., Korea, 23.IV.1983, Y. B. Cho; 4♂♂ 2♀♀, Jeju Prov., Korea, 22.VII.1985, Y.B. Cho (1♂ 1♀, on slide); 1♀, Donnaeko, Sanghyo-dong, Seogwipo-city, Jeju Prov., Korea, 18.X.1985, K.-S. Lee; 1♀, Donnaeko, Sanghyo-dong, Seogwipo-city, Jeju Prov., Korea, 22.X.1985, K.-S. Lee; 1♀, Jeongbangpolpo-waterfall, Donghong-dong, Seogwipo-city, Jeju Prov., Korea, 29.X.1985, K.-S. Lee; 1♂, Gasi-ri, Pyoseon-myeon, Namjeju-gun, Jeju Prov., Korea, 5.V.1985, K.-S. Lee; 2, Japan, G. Lewis, 1910–320, Nagasaki, 22.V–3.VI.81; 2, Japan, G. Lewis, 1910–320, Kumamoto, 23.IV–26.IV.81; 3, Japan, G. Lewis, 1905–313 (1, Holotype of Aleochara japonica Sharp, deposited in the Natural History Museum, London); 1, Japan, G. Lewis, 1910–320.

##### Distribution.

China, India, Japan, Korea, Nepal, Taiwan (see Smetana, 2004: 356).

##### Remarks.

This species is a new record for the Korean peninsula.

#### Aleochara (Xenochara) intricata

Mannerheim

Fig. 2

Aleochara intricata Mannerheim 1830: 96; Fenyes 1920: 404; Bernhauer and Scheerpeltz 1926: 782; Portevin 1929: 236; Palm 1972: 428; Lohse 1974: 296;  Welch 1997: 26; Smetana 2004: 354; Assing 2007a: 60; 2007b: 184.
Aleochara terminata Stephens 1832: 158.
Aleochara celer Stephens 1832: 161.
Aleochara biguttata Heer 1839: 315.
Aleochara croatica Penecke 1901: 12.

##### Redescription.

Length 3.5–6.0 mm. Body large and robust; brownish black; antennomeres 1–3 and legs brown; elytra yellow to yellowish brown and bicolored. Antennomeres 1–3 elongate, 4 longer than wide, 5–8 weakly transverse and 9–10 transverse. *Mouthparts*. Labrum transverse, bearing approximately 8 small, setae, and approximately 26 long setae, a-seta, b-seta, and pores present; b-seta rounded apically (arrow indicates b-seta, Fig. 2c). Labium with pseudopores in median area; approximately 3 real pores and pseudopores present in lateral area; pair of basal pores present (Fig. 2d). Ligula with approximately 4 pairs of small setae apically (Fig. 2d). Labial palpi with large a-, b-, and f-seta of 12 setae (a–h, α–δ) present; β-seta close to twin pores (tp); d- and c-seta at same level (Fig. 2e). Mentum transverse, bearing 4 pairs of main setae (b, u, v, w), and 4 extra setae, and pores present (Fig. 2f). *Thorax*. Mesoventrite completely carinate (arrow, Fig. 2a). Elytra with round latero-posterior margin. *Abdomen*. Abdominal tergite VIII with many short setae and pores; posterior margin weakly emarginate (Fig. 2g). Abdominal sternite VIII with many short setae and pores; posterior margin rounded (Fig. 2h, i). *Genitalia*. Median lobe as in Figs 2k and l. Paramere without fovea in hinge zone (Fig. 2j). Spermatheca as in Fig. 2m.

##### Material examined.

7, Hol-ri, Ganseong-eup, Goseong-gun, Gangwon Prov., Korea, 31.VIII–1.IX.1984, Y.-S. Kim (2♂♂2♀♀, on slide); 1, Gohan-ri, Sabuk-eup, Gangwon Prov., Korea, 27.IV.1985, J.-I. Kim; 1, Deakwan-ryeong, Gangwon Prov., Korea, 28.VI.1984, Y.-S. Kim; 2, Oigapyeong, Inje-gun, Gangwon Prov., Korea, 26.V.1986, Y.-S. Kim; 1, Baekdamsa-temple, Inje-gun, Gangwon Prov., Korea, 26.V.1986, Y.-S. Kim; 1, Dammaeul, Cheongsong, Gyeongbuk Prov., Korea, 24.VI.1988, K.-S. Jang; 1, Tonghan, Anjeong, Jeonbuk Prov., Korea, 5.VI.1988, Y.-S. Kim; 6, Seilles, carriere 1, 11/19 VIII 1945, G. Fagel; 2, Anseremme car, vers Freyr, 26 VIII 1946, G. Fagel; 2, Abruzzo, A. colomba, Italy, 26.VII.1894, P Fiori; 12, Lazio, Roma, Lirezzi, Italy, A. Fiori.

##### Distribution.

Korea, Asia, Europe, North Africa (see Smetana, 2004: 354).

##### Remarks.

This species is a new record for South Korea.

#### Aleochara (Xenochara) peninsulae

Bernhauer

Fig. 3

Aleochara peninsulae Bernhauer 1936: 325; Smetana 2004: 360.

##### Holotype examined.

Male mounted on card, with mouthparts, aedeagus and abdominal apex (segment VIII+) mounted in balsam on two transparent cards, labeled as follows: “Shimabara Unzen 2200F 2. 8. 34. Suenson” [printed]; “peninsulae Brnh. Typus un. Polychara” [handwritten yellow label]; “peninsulae Brnh. Typus unic.” [handwritten white label]; “Chicago NHMus M. Bernhauer Collection” [printed]; “HOLOTYPE Aleochara peninsulae Bernhauer, 1936 teste Park & Ahn 2007” . Deposited in the Field Museum of Natural History (FMNH), Chicago, USA.

##### Redescription.

Length 3.8–5.4 mm. Body compact and robust; reddish black; antenna, elytra, and legs brownish black; elytra bicolored. Antennomeres 1–3 elongate, 4 longer than wide, 5 subquadrate, and 6–10 transverse. *Mouthparts*. Labrum transverse, bearing approximately 8 small, setae, and approximately 19 long setae, a-seta, b-seta and pores present; b-seta acute (arrow indicates b-seta, Fig. 3c). Labium with pseudopores in median area; approximately 3 pores and pseudopores present in lateral area; a pair of distal setae present (arrow indicates distal seta, Fig. 3d). Ligula with approximately 3 pairs of small setae apically (Fig. 3d). Labial palpi with large a-, b-, and f-seta of 12 setae present (a–h, α–δ); β-seta close to twin pores (tp); d-seta higher than c-seta (Fig. 3e). Mentum transverse, bearing 4 pairs of main setae (b, u, v, w), and approximately 2 extra setae, and pores present (Fig. 3f). *Thorax*. Mesoventrite completely carinate (arrow, Fig. 3a). Elytral latero-posterior margin emarginate. *Abdomen*. Abdominal tergite VIII with many short setae and pores; posterior margin emarginate; undulate on male (Fig. 3g, h). Male abdominal sternite VIII with many short setae and pores; posterior margin produced on male (arrow, Fig. 3i) and rounded on female (Fig. 3j). *Genitalia*. Median lobe as in Figs 3l and m. Paramere with fovea in hinge zone (arrow, Fig. 3k). Spermatheca as in Fig. 3n.

##### Material examined.

2, Gyeongsang Univ., Gajoa-Dong, Jinju-city, Gyeongnam Prov., Korea, 26.III.2003, C.-S. Lim, *ex* bait trap; 2, Gyeongsang Univ., Gajoa-Dong, Jinju-city, Gyeongnam Prov., Korea, 2 VI 2003, C.-S. Lim, *ex* bait trap; 1, Gyeongsang Univ., Gajoa-Dong, Jinju-city, Gyeongnam Prov., Korea, 27.III.2003, C.-S. Lim, *ex* bait trap; 2, Gyeongsang Univ., Gajoa-Dong, Jinju-city, Gyeongnam Prov., Korea, 22.III.2003, C.-S. Lim, *ex* bait trap; 1, Gyeongsang Univ., Gajoa-Dong, Jinju-city, Gyeongnam Prov., Korea, 12.III.2003, C.-S. Lim, *ex* bait trap; 1, Gyeongsang Univ., Gajoa-Dong, Jinju-city, Gyeongnam Prov., Korea, 7.IV.2003, C.-S. Lim, *ex* bait trap; 1, Gyeongsang Univ., Gajoa-Dong, Jinju-city, Gyeongnam Prov., Korea, 15.III.2003, C.-S. Lim, *ex* bait trap; 1, Gyeongsang Univ., Gajoa-Dong, Jinju-city, Gyeongnam Prov., Korea, 24.III.2003, C.-S. Lim, *ex* bait trap; 1, Gyeongsang Univ., Gajoa-Dong, Jinju-city, Gyeongnam Prov., Korea, 29.V.2003, C.-S. Lim, *ex* bait trap; 1, Gyeongsang Univ., Gajoa-Dong, Jinju-city, Gyeongnam Prov., Korea, 30.III.2003, C.-S. Lim, *ex* bait trap; 1, near 1100m Rest Area, Jeju Prov., Korea, 30.V–17.VI.2003, Y.-B. Cho, *ex* bait trap.

##### Distribution.

Japan, Korea.

##### Remarks.

This species is a new record for the Korean peninsula.

#### Aleochara (Xenochara) puberula

Klug

Fig. 4

Aleochara puberula Klug 1832: 139; Ganglbauer 1895: 32; Fenyes 1920: 403; Bernhauer and Scheerpeltz 1926: 781; Portevin 1929: 236; Palm 1972: 426; Lohse 1974: 296; Klimaszewski 1984: 46; Smetana 2004: 360.
Aleochara vaga Erichson 1839: 172.
Aleochara deserta Erichson 1839: 173.
Aleochara decorata Aubé 1850: 311.
Aleochara armitagei Wollaston 1854: 559.
Aleochara badia Motschulsky 1858: 237.
Oxypoda sanguinolenta Motschulsky 1858: 241.
Oxypoda brunnescens Motschulsky 1858: 243.
Aleochara dubia Fauvel 1863: 428.
Oxypoda analis MacLeay 1873: 135.
Baryodma bipartita Casey 1894: 287.
Aleochara major Eichelbaum 1912: 176.
Aleochara puberula  See Klimaszewski (1984) for additional synonymies and references.

##### Redescription

Length 3.5–5.5 mm. Body reddish brown; antennomeres 1–3, elytra, and legs brown; elytra bicolored. Antennomeres 1–3 elongate, 4 longer than wide, 5 subquadrate, and 6–10 transverse. *Mouthparts*. Labrum transverse, bearing approximately 11 small, setae, and approximately 19 long setae, a-seta, b-seta, and pores present; b-set a rounded at tip (arrow indicates b-seta, Fig. 4b). Labium with pseudopores in median area; approximately 3 real pores and pseudopores present in lateral area; a pair of basal pores present (Fig. 4c). Ligula with approximately 2 pairs of small setae apically (Fig. 4c). Labial palpi with large a-, b-, and f-seta of 12 setae present (a–h, α–δ); β-seta close to twin pores (tp); c-seta higher than d-seta (Fig. 4d). Mentum transverse, bearing 4 pairs of main setae (b, u, v, w), and 4 extra setae, and pores present (Fig. 4e). *Thorax*. Elytral latero-posterior margin emarginate. *Abdomen*. Abdominal tergite VIII with many short setae and pores; posterior margin weakly emarginate (Fig. 4f). Abdominal sternite VIII with many short setae and pores; apical margin rounded on male (Fig. 4g) and truncated on female (Fig. 4h). *Genitalia*. Median lobe as in Figs 4j and k. Paramere with fovea absent in hinge zone (Fig. 4i). Spermatheca coiled in duct (Fig. 4l).

##### Material examined.

2, Seoguipo-city, Jeju Prov., Korea, 18.VI.1985, K.-S. Lee; 1, Anseong, Muju-gun, Jeonbuk Prov., Korea, 5.VI.1988, G.-S. Jang; 22, Reunion 22–23.I.1992, Ravine de St. Gilles Bassin Cormoran, J. Janaj lgt; 1, Philippinen, Manila, Luy, 2.XI.1914; 1, Argentina, Prov. Tucuman, 450m, I 1905, Steinbach; 1, N. Palawan, Bakuit, 12.XI–22.XII 1913, Bottcher; 4, Sud algérien: Mrhaier 120 Km S de Biskra, 14.V.1954, G. Fagel; 4, China, B. M. 1980–491, P. M. Hammond, Guangdong, Guangzhou, Baiyunshan, 27.IX.80; 4, China, B. M. 1980–491, P. M. Hammond, Guizhou, 20m, S. Guilin, 22.IX.80; 1, Japan, Honshu, B. M. 1980–492, P. M. Hammond, Nara, ft of Mt. Kasuga, 20.VIII.80; 2, Japan, Sharp Coll., 1905–313; 4, Japan, G. Lewis, 1910–320.

##### Distribution.

Japan, Korea, Asia, Europe, North Africa, North America (see Smetana, 2004: 360).

#### Aleochara (Xenochara) tristis

Gravenhorst

Fig. 5

Aleochara tristis Gravenhorst 1806: 170; Mulsant and Rey 1874: 72; Fowler 1888: 14; Ganglbauer 1895: 34; Fenyes 1920: 405; Bernhauer and Scheerpeltz 1926: 784; Portevin 1929: 237; Palm 1972: 428; Lohse 1974: 296; Klimaszewski 1984: 37; Welch 1997: 26; Smetana 2004: 361.
Staphylinus bipunctata Olivier 1795: 31.
Staphylinus geometrica Schrank 1798: 642.
Aleochara flavomaculata Ménétriés 1832: 147.
Aleochara bimaculata Stephens 1832: 158.
Aleochara nigripes Miller 1853: 27.
Aleochara erectesetosa Jekel 1873: 41.
Baryodma nigripennis Mulsant and Rey 1874: 76.
Aleochara tristis  See Klimaszewski (1984) for additional synonymies and references.

##### Redescription.

Length 3.7–6.4 mm. Body black; antenna and legs reddish black; elytra yellow to yellowish brown and bicolored. Antennomeres 1–3 elongate, and 4–10 transverse. *Mouthparts*. Labrum transverse, bearing approximately 9 small, setae, and approximately 17 long setae, a-seta, b-seta, and pores resent; b-seta acute (arrow indicates b-seta, Fig. 5b). Labium with pseudopores in median area; about 3 real pores and pseudopores present in lateral area; a pair of distal setae present (arrow indicates distal seta, Fig. 5c). Ligula with approximately 3 pairs of small setae apically (Fig. 5c). Labial palpi with large a-, b-, and f-seta of 12 setae present (a–h, α–δ); β-seta close to twin pores (tp); c- and d-seta same level (Fig. 5d). Mentum transverse, bearing 4 pairs of main setae (b, u, v, w), and 13 extra setae, and pores present (Fig. 5e). *Thorax*. Elytra with round latero-posterior margin. *Abdomen*. Abdominal tergite VIII with many short setae and pores; posterior margin emarginate (Fig. 5f). Abdominal sternite VIII with many short setae and pores; apical margin rounded (Fig. 5g, h). *Genitalia*. Median lobe with coiled flagellum (arrow, Figs 5j and k). Paramere with fovea in hinge zone (arrow, Fig. 5i). Spermathecal duct coiled (Fig. 5l).

##### Material examined

1, Russia, Tadshikistan, 1981, Duechanbe env, on light, 16–18.VI.1981, K. Majer; 36, Russia, Tadshikistan, Umg. Duechanbe env, 800–1200m, 4–14.IX.1983, U. Arnold.

##### Distribution.

Korea, Asia, Europe, North Africa, North America (see Smetana, 2004: 361).

##### Remarks.

See Klimaszewski (1984: 39) for reason why the older names Staphylinus bipunctata Olivier or Staphylinus geometrica Schrank do not have priority. We used Russian specimens for the redescription.

## Supplementary Material

XML Treatment for Xenochara
